# Editorial: Apoptosis Induction/Suppression: A Feasible Approach for Natural Products to Treatment of Diseases, Volume II

**DOI:** 10.3389/fphar.2022.923562

**Published:** 2022-05-16

**Authors:** Wei Peng, Xin Luan, Hong Zhang

**Affiliations:** ^1^ School of Pharmacy, Chengdu University of Traditional Chinese Medicine, Chengdu, China; ^2^ Institute of Interdisciplinary Integrative Medicine Research, Shanghai University of Traditional Chinese Medicine, Shanghai, China

**Keywords:** natural product, apoptosis, cancers, tumors, volume II

## Introduction

Apoptosis is the predominant form of programmed cell death, which is crucial for the normal cell development, organ growth and tissue homeostasis in organisms. In physiological conditions, the cell death and proliferate keep in a dynamic balance. In contrary, the unbalance of cell death and proliferation would result in lots of serious diseases, such as cancers, Alzheimer’s disease, Parkinson’s disease, and stroke, etc. Similar to the previous Volume I, the research topic “***Apoptosis Induction/Suppression: A Feasible Approach for Natural Products to Treatment of Diseases, Volume II***” provide an academic platform to discuss the novel signal molecules for the apoptosis-related signal pathway, and how natural products can be used to treat various diseases mentioned above via induction or suppression of apoptosis.

## Natural Products Induce Apoptosis in Tumors

Similar to Volume I of our topic for “***Apoptosis Induction/Suppression: A Feasible Approach for Natural Products to Treatment of Diseases***”, most of the papers in Volume II suggested that inducing apoptosis is one of the predominant molecular mechanisms for natural products to treat cancers, such as lung, liver, colorectum, prostate, and breast cancers, *etc*.

It is known that lung cancer is the leading cause of cancer caused death worldwide. Ding et al. reported two natural sesquiterpene lactones named alantolactone and brevilin A attenuate the paclitaxel resistance induced pro-apoptotic effect in the paclitaxel-resistant A549 lung cancer cells. In addition, it is reported that natural products could be also used to treat another type of malignant tumor with promising prospect. Jiang et al. reviewed the antitumor effect of secondary metabolites from Rhei Radix et Rhzoma against liver cancer, which, including emodin, rhein, physcion, aloe-emodin, gallic acid, and resveratrol, could suppress liver cancer via induction of apoptosis owing to their multi-targets and multi-pathway characteristics. Su et al. reported the anti-cancer activity of acidic polysaccharides isolated from the *Scutellaria barbata* D. Don, which could induce apoptosis in liver cancer cells via regulation of the apoptosis related proteins such as P53, Bax, Bcl-2 ratio, and cell cycle related proteins of cyclin D1 and CDK4. Sempervirine is an alkoloid isolated from *Gelsemium elegans* (G. elegans) Benth. considered as a toxic plant. Yue et al. reported the anticancer effects of sempervirnine on liver cancer *in vivo* and *in vitro*, and the potential molecular mechanisms are related to promote apoptosis via regulating the Wnt/β-catenin pathway. Besides, Wan et al. reported that ascorbic acid could suppress the growth and metastasis of liver cancer via promoting apoptosis of the cells by regulation of stemness genes.

Breast cancer is the most common malignancy in women with high morbidity and mortality, which is serious harm to the health of women, especially the subtype of triple negative breast cancer (TNBC). Yuan et al. present a review on the anticancer effects of natural compounds against breast cancer. The authors indicated that natural product could induce the apoptosis in breast cancer cells via multiple pathways, including mitochondria, FasL/Fas, PI3K/Akt, ROS, and MAPK. Maackiain is a natural compound isolated from the *Spatholobus suberectus* Dunn., which possesses significant anticancer effect against TNBC via induction of apoptosis and suppression of foci formation, migration and invasion of the cells by modulating miR-374a/GADD45A axis (Peng et al.). Besides, Zhu et al. present a review regarding berberine, a known natural compound isolated from the *Coptis chinensis* Franch., which possesses wide spectrum anticancer activities against lung cancer, liver cancer, and breast cancer, *etc*.

Similar to liver cancer, colorectal cancer is another type of cancer with high incidence, especially in China. Safflower polysaccharide is an active fraction isolated from the known traditional Chinese medicine of *Carthamus tinctorius* L. Wang et al. found that the safflower polysaccharide could induce apoptosis in colorectal cancer cells through macrophage polarization. Chaetocin is a natural product produced by a species of fungi of Chaetomium, which has anticancer effects against various type of cancers. Wang et al. investigated the anticancer effect of chaetocin on colorectal cancer, and found that this natural compound can induce apoptosis in colorectal cancer cells via regulation of ROS/JNK/C-Jun pathway. Prostate cancer is a commonly diagnosed malignant cancer in men, which does serious harm to the health of men. Bai et al. present a review regarding the anticancer effects and molecular mechanisms of natural products against prostate cancer. The authors suggested that natural products including extracts and monomers possess promising anticancer effects against prostate cancer, and the molecular mechanisms are related to induction of apoptosis. Chang et al. extracted the total flavonoids of Litchi seeds (TFLS), and studied their anticancer effect on prostate cancer *in vivo* and *in vitro*. The related results showed that TFLS induced the apoptosis and phenotypic reversal of EMT via inhibiting Akt/mTOR and NF-κB signaling pathways. Furthermore, Li et al. summarized the anticancer effects of natural products on pancreatic cancers via induction of apoptosis. Li et al. reported the anticancer effect of borneol against glioma related to induction of apoptosis and autophagy. Besides, another review by Li et al. suggested that natural products are promising therapeutics for treating cancers via induction of apoptosis and inhibition of angiogenesis.

## Natural Products Inhibit Apoptosis to Treat Other Diseases

Excessive apoptosis is the main reason for some diseases, such as ischemic heart disease (IHD), bone loss related diseases, and organs injury, *etc*. IHD is commonly caused by insufficient blood flow to the cardiac tissues, which has high morbidity and mortality. Chen et al. summarized the protective effects of ginsenoside on the myocardial ischemia/reperfusion injury (MIRI), and suggested that the potential molecular mechanisms are correlated to inhibition of myocardial cell apoptosis. Furthermore, Tang et al. reported that the colchicine, which is an alkaloid from *Colchicum autumnale*, can protect H_9_C_2_ cells from hypoxia/reoxygenation injury via anti-apoptosis by activating the PI3K/Akt/eNOS pathway. Besides, using network analysis, Duan et al. found the *Yi-Qi-Tong-Luo*-Capsule alleviates the myocardial ischemia by suppression of the oxidative stress induced apoptosis through the PI3K/Akt/Nrf2 pathway. Peng et al. reported the ginsenoside Rb1 attenuates the triptolide induced cell apoptosis in human normal liver cell line of HL-7702 cells by regulation of the Keap1/Nrf2/ARE signaling pathway. Chen et al. reported the curcumin could protect the rat tracheal epithelial cells from cigarette smoke extract induced apoptosis. Pyxinol is one of the active compounds from *Lichenes*. Yang et al. found that this compound could reduce the cisplatin induced nephrotoxicity via ameliorating DNA damage response. In addition, Fu et al. reported the *Periplaneta americana* L. extract has gastroprotective effects on ethanol-induced gastric ulcer in mice via inhibition of apoptosis related pathways. The apoptosis of intestinal epithelial cells (IECs) plays a crucial role in disease development of Ulcerative colitis (UC). Liu et al. present a review to summary the anti-apoptotic effects of natural products on IECs. Long-term abuse of methamphetamine could cause neurodegenerative changes in central dopaminergic neurons. Zeng et al. reviewed the protective effects of natural products against methamphetamine induced neuronal apoptosis and their potential molecular mechanisms. Besides, Almohaimeed et al. reported the extracts from *Cucurbita pepo* L. effectively ameliorate the chronic stress-induced behavioral, biochemical, and adrenal structural changes mostly through anti-apoptosis, antioxidant and anti-inflammatory effects.

Besides these mentioned above, the investigation by Wang et al. indicated that induction of PI3K/Akt related osteoclasts apoptosis plays an important role for *Buxue Tongluo* Pills to treat osteonecrosis of the femoral head. In addition, Rao et al. suggested the *Fengreqing Oral Liquid* exerts anti-inflammatory effects via promoting apoptosis through inhibiting PI3K/Akt and NF-κB signaling pathways.

We believe that this topic shows the latest studies regarding treatment of various diseases by natural products via regulating apoptosis from fundamental theory and experiments *in vitro* and *in vivo*. Based on these works, it is suggested that apoptosis induction/suppression is helpful for treating various diseases, including cancers, ischemia-reperfusion (I/R) injury related diseases, bone loss related diseases, and organs injury, *etc*. However, similar to the previous Volume I, some other important apoptosis related diseases have not been mentioned in Volume II, such as Alzheimer’s disease, Parkinson’s disease, and rheumatoid arthritis, etc. Therefore, more works could be devoted to finding novel natural agents with pharmacological activities against these diseases via apoptosis induction/suppression.

